# Multiplex PCR and Next Generation Sequencing for the Non-Invasive Detection of Bladder Cancer

**DOI:** 10.1371/journal.pone.0149756

**Published:** 2016-02-22

**Authors:** Douglas G. Ward, Laura Baxter, Naheema S. Gordon, Sascha Ott, Richard S. Savage, Andrew D. Beggs, Jonathan D. James, Jennifer Lickiss, Shaun Green, Yvonne Wallis, Wenbin Wei, Nicholas D. James, Maurice P. Zeegers, KK Cheng, Glenn M. Mathews, Prashant Patel, Michael Griffiths, Richard T. Bryan

**Affiliations:** 1 Institute of Cancer & Genomic Sciences, University of Birmingham, Birmingham, B15 2TT, United Kingdom; 2 Warwick Systems Biology Centre, University of Warwick, Coventry, CV4 7AL, United Kingdom; 3 West Midlands Regional Genetics Laboratory, Birmingham Women’s Hospital NHS Foundation Trust, Birmingham, B15 2TG, United Kingdom; 4 Clinical Trials Unit, University of Warwick, Coventry, CV4 7AL, United Kingdom; 5 Department of Complex Genetics, NUTRIM School of Nutrition and Translational Research in Metabolism, Maastricht University Medical Centre, Maastricht, The Netherlands; 6 School of Health and Population Sciences, University of Birmingham, Birmingham, B15 2TT, United Kingdom; 7 Warwick Medical School, University of Warwick, Coventry, CV4 7AL, United Kingdom; Centro Nacional de Investigaciones Oncológicas (CNIO), SPAIN

## Abstract

**Background:**

Highly sensitive and specific urine-based tests to detect either primary or recurrent bladder cancer have proved elusive to date. Our ever increasing knowledge of the genomic aberrations in bladder cancer should enable the development of such tests based on urinary DNA.

**Methods:**

DNA was extracted from urine cell pellets and PCR used to amplify the regions of the *TERT* promoter and coding regions of *FGFR3*, *PIK3CA*, *TP53*, *HRAS*, *KDM6A* and *RXRA* which are frequently mutated in bladder cancer. The PCR products were barcoded, pooled and paired-end 2 x 250 bp sequencing performed on an Illumina MiSeq. Urinary DNA was analysed from 20 non-cancer controls, 120 primary bladder cancer patients (41 pTa, 40 pT1, 39 pT2+) and 91 bladder cancer patients post-TURBT (89 cancer-free).

**Results:**

Despite the small quantities of DNA extracted from some urine cell pellets, 96% of the samples yielded mean read depths >500. Analysing only previously reported point mutations, *TERT* mutations were found in 55% of patients with bladder cancer (independent of stage), *FGFR3* mutations in 30% of patients with bladder cancer, *PIK3CA* in 14% and *TP53* mutations in 12% of patients with bladder cancer. Overall, these previously reported bladder cancer mutations were detected in 86 out of 122 bladder cancer patients (70% sensitivity) and in only 3 out of 109 patients with no detectable bladder cancer (97% specificity).

**Conclusion:**

This simple, cost-effective approach could be used for the non-invasive surveillance of patients with non-muscle-invasive bladder cancers harbouring these mutations. The method has a low DNA input requirement and can detect low levels of mutant DNA in a large excess of normal DNA. These genes represent a minimal biomarker panel to which extra markers could be added to develop a highly sensitive diagnostic test for bladder cancer.

## Introduction

Flexible cystoscopy, combined with urine cytology, is the gold standard approach for detecting bladder tumours. Long-term surveillance for recurrence post-treatment is both burdensome for patients and expensive for healthcare providers. It has long been hoped that a test based on molecules released into the urine by tumour cells might reduce reliance on cystoscopy, however, to date sufficiently sensitive and specific tests have not been developed and adopted by clinicians [1].

Existing urinary biomarkers for bladder cancer such as NMP22 and BTA lack sensitivity for low-grade disease and may be falsely elevated due to non-malignant conditions and haematuria [1]. Great efforts have been expended on discovering urinary biomarkers for the non-invasive detection of bladder cancer with particular success being achieved measuring tumour specific nucleic acid variants [2,3,4]. Recent genomic and transcriptomic experiments have shown that low-grade NMIBC and CIS/HG-NMIBC/MIBC have distinct mutation and gene expression profiles and that even within NMIBC considerable heterogeneity exists (reviewed in [5]). Thus, it seems likely that a panel of biomarkers which captures the diversity of UBC will be required to reliably detect disease.

Urine tests based on DNA show considerable promise for the non-invasive detection of UBC. In theory, if tumour DNA is present, it can be amplified by PCR and cancer-specific alterations detected. Two of the most frequently mutated genes in bladder cancer with point-mutation hotspots are *FGFR3* [6] and *TERT* [7,8,9,10]. Both have been assessed as biomarkers for detecting bladder cancer in urinary DNA in separate studies [7,11,12], but they have not been combined in a NGS-based assay. The *TERT* promoter is mutated in approximately 65% of bladder tumours regardless of stage and grade [13] and represents the best single biomarker for bladder cancer with a recent report of 62% sensitivity at 90% specificity for detecting primary bladder tumours [7].

We have now developed an assay which uses high-thoughput deep-sequencing of the regions of 6 UBC associated genes which contain mutation hotspots and analysed urinary DNA from 232 patients to evaluate it’s ability to non-invasively detect UBC. Theoretically, deep sequencing should reliably detect even very low levels of tumour DNA in a large excess of non-tumour DNA. Furthermore, this technology is maturing to the point where it is becoming a routine approach in clinical genetic testing laboratories.

## Materials and Methods

### Ethics statement

All samples were obtained after written informed consent and approval by the appropriate UK national research ethics review boards (National Research Ethics Service references stated below); signed consent forms were held within individual patient files, with consent documented in the relevant study databases.

The UBC and non-UBC patients were collected as part of the Bladder Cancer Prognosis Programme, BCPP (NRES Committee East Midlands—Derby: 06/MRE04/65), which incorporated prospective biospecimen collection for biomarker research.

A second urine collection (NRES Committee North West–Haydock: 15/NW/0079) was undertaken from patients undergoing surveillance following TURBT for NMIBC.

### Patients

Urine was collected from three groups of patients: ‘non-UBC’, ‘UBC’ and ‘post-UBC’. The UBC and non-UBC patients were collected as part of the West Midlands’ Bladder Cancer Prognosis programme, 2005–11 (BCPP, described in detail here: [14]). Briefly, midstream urine (20–50 ml) was collected at the time of diagnosis from patients with cystoscopic findings indicative of primary bladder cancer; urine was centrifuged and pellet and supernatant stored at -80°C. After sample collection, each patient underwent TURBT and definitive diagnosis by histopathological examination of the resected tissue. Since primary bladder *carcinoma in situ* (CIS) is a rare lesion in the BCPP cohort and elsewhere [15], patients with primary CIS were excluded as we did not have sufficient numbers to be able to draw valid conclusions. Some patients recruited to BCPP and who gave urine samples were subsequently diagnosed with non-malignant urological conditions, and are included in the study as ‘non-UBC’ patients.

Separately, a second prospective urine collection was undertaken in the West Midlands during 2012 from an independent group of 91 patients undergoing surveillance following TURBT for NMIBC, and utilising the same urine collection protocol. With the exception of 2 patients, all patients were disease-free on the date of urine collection (‘post-UBC’ group). The 2 patients with recurrent disease were added to the UBC group. Subsequent cystoscopy data were available for 38 of the 89 ‘post-UBC’ patients, and showed no further recurrences at a mean of 8 months. DNA was extracted from urine pellets using urine DNA isolation kits for exfoliated cells and bacteria (NorgenBiotek.com) and eluted in 100 μl of deionised water.

### PCR and barcoding

A single multiplex PCR amplification was used to amplify 16 amplicons in the *TERT* promoter and coding regions of *FGFR3*, *PIK3CA*, *TP53*, *HRAS*, *RXRA* and *KDM6A* (primer sequences in S1 Table). The PCR method was adapted from Allory *et al* [7] as the *TERT* promoter region is difficult to amplify and initial attempts using several other polymerases failed. PCR amplifications consisted of 9 μl DNA, 10 μl KAPA2G Robust DNA Polymerase (KapaBiosystems) and 1 μl of primers (0.2 μM final for each primer). The PCR program was: 95°C for 3 minutes, 35 x 95°C for 15s, 60°C for 15s, 72°C for 15s followed by 3 min at 72°C (primer sequences are provided in Supporting Information, S1 Table). The PCR products were diluted 1/100 with deionised water and 10 bp barcodes added by a second 15 cycle PCR using KAPA2G Robust DNA Polymerase and single direction access array barcodes for Illumina sequencers (Fluidigm).

### Sequencing and data analysis

The barcoded PCR products were mixed and cleaned using AMPure beads. The pooled amplicons were then mixed 7:3 with PhiX (Illumina), denatured and clustered at 6 pM on a Miseq 500 cycle flow-cell and sequenced (250 cycles forward, 10 cycles barcode, 250 cycles reverse). Raw trace files were processed with cutadapt (version 1.8.1, Python 2.6.6) in paired end mode to remove adapter sequences, and to filter out pairs with a sequence < 100 nt to exclude short read artefacts. Local alignments of reads to the hg19 genome were performed using bowtie2 (version 2.2.4) in paired end mode. SAM alignment files were converted to BAM files, sorted and indexed using samtools (version 0.1.19). BAM files were processed with bam-readcount (https://github.com/genome/bam-readcount), <minimum base quality threshold set to 0|30>, to generate position-specific nucleotide metrics within the co-ordinates of the amplified loci. Bam-readcount outputs were processed with a custom written Perl script. Mutations were called where a non-reference base was present in >3 reads and at a frequency of between 2.5–97.5% in both directions. We limited our analysis to single base substitutions previously reported in UBC in COSMIC.

### Sanger sequencing

Following PCR utilising the same primers and same BCPP urine samples as for NGS, Sanger sequencing on an ABI 3730 sequencer was performed to provide orthogonal confirmation of the presence and absence of *TERT* and *FGFR3* mutations in 12 and 8 samples, respectively.

### Data availability

All the data necessary for replication are within the manuscript. The PCR primer sequences are shown in S1 Table and we have used percentage of mutant reads as the input for all graphs, sensitivities, specificities, etc, and these are presented for every locus in every sample in S2 Table.

## Results

### Optimisation of NGS-amplicon sequencing on urine DNA

When tested individually, all 16 primer pairs generated the correct size products. The primers were then combined and the multiplex PCR tested over a range of template concentrations (designed to mimic the variable amount of DNA obtained from urine pellets): the PCR worked with the lowest DNA input tested (0.5 ng) and the amount of product generated was independent of the amount of DNA template between 2 and 50 ng (50 ng being the highest input tested). In this experiment, next generation sequencing of the multiplex PCR products gave an average of 6000 reads per amplicon; even for samples where less than 1 ng of DNA was used, read depths averaged >4000 (S1 Fig). Titration of DNA extracted from the bladder cancer cell lines MGH-U3 (*FGFR3* mutant) and SW-780 (*TERT* promoter mutant) into wtDNA demonstrated that the percentage of mutant reads reliably reflected the percentage of mutant DNA down to levels below the 2.5% threshold used here (S2 Fig). Furthermore, the analysis of germline DNA from 6 BCPP patients without UBC demonstrated a mean ‘false positive’ read frequency of 0.20% ± 0.28 at the mutation loci described below.

Water blanks (n = 37) were run in randomly assigned wells alongside the urine DNA samples to test for contamination that could generate false results, particularly for urine samples with low DNA concentrations. The mean read depth in the water blanks was 160 (less than 3% of the mean read depth in the samples). Samples with low DNA concentrations and yielding a mean read depth of <500 were excluded from the study. This lead to the exclusion of 10 samples out of 242 i.e. the assay worked successfully in 96% of the samples. Patient information for the remaining 232 patients is shown in Table 1.

**Table 1:** Patient demographics. NA: not applicable, NK: not known.

	n	Grade (G1/G2/G3)	Mean Age (years)	Gender (M/F)
**Non-UBC**	20	NA	68.1	17/3
**Post-UBC**	89	NA	68.9	60/29
**UBC pTa**	41	14/23/4	68.5	32/9
**UBC pT1**	40	1/13/26	70.0	30/10
**UBC pT2+**	39	0/3/36	74.3	25/14
**UBC (recurrent)**	2	NK	80.5	1/1

### Detection of mutations in urinary DNA

**Fig 1:**
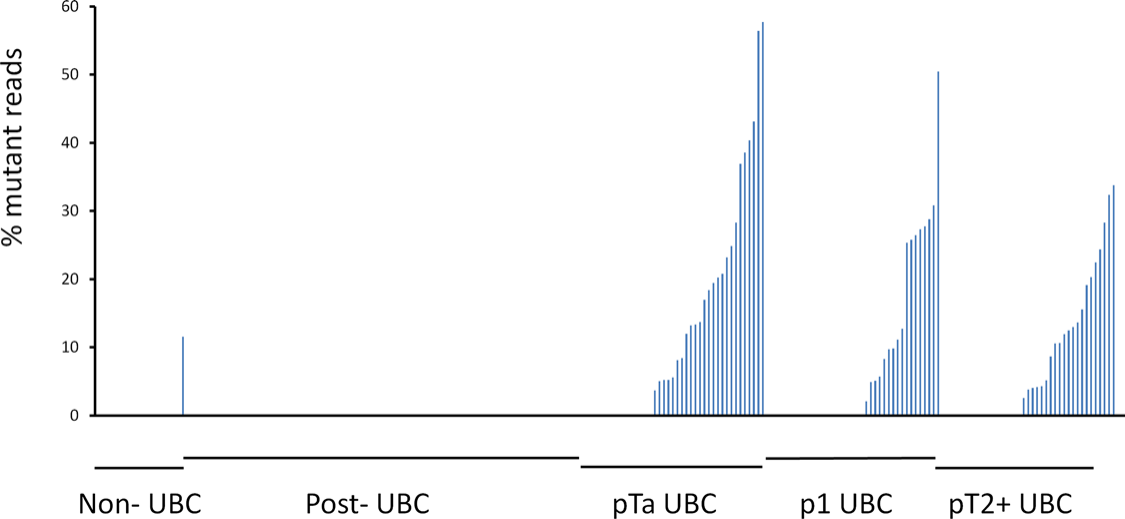
Frequency of mutant *TERT* promoter reads in urinary DNA. The graph shows the percentage of mutant reads in each patient sample considering point mutations at positions chr5:1295228, chr5:1295242/1295243 and chr:1295250.

**Fig 2:**
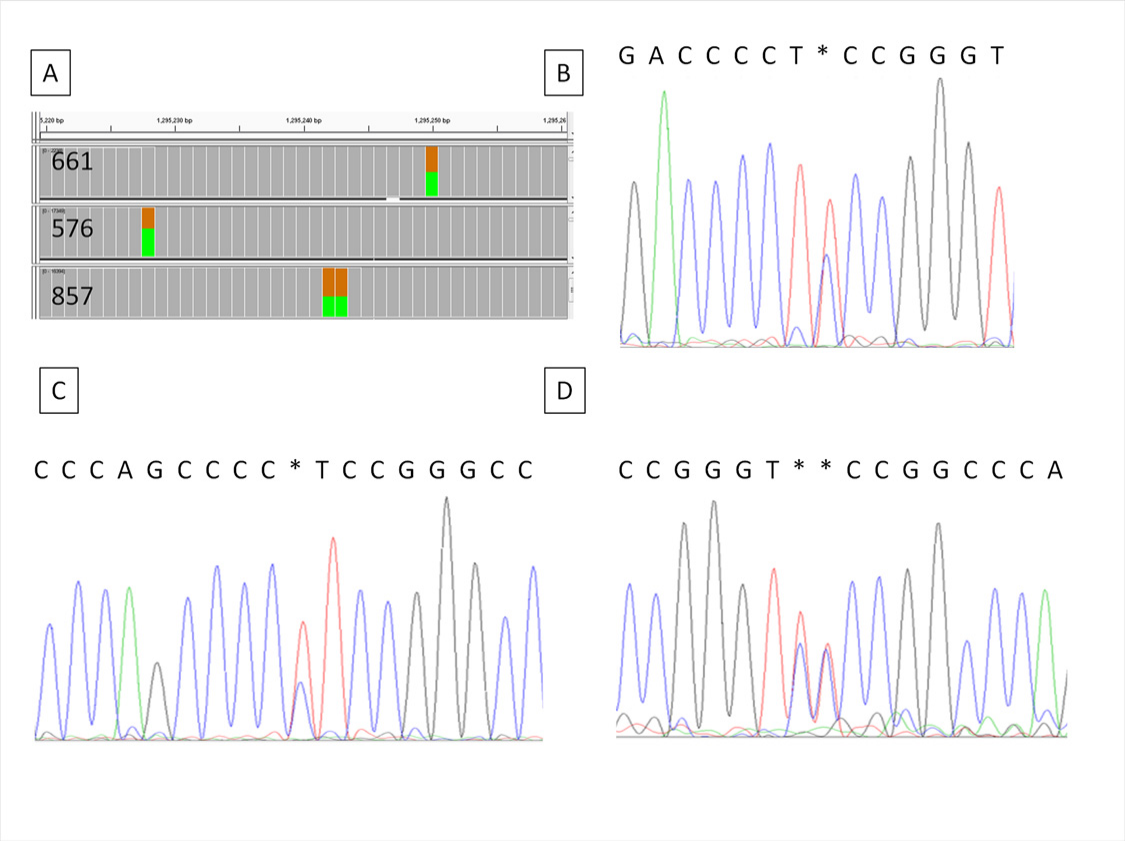
Confirmation of *TERT* mutations by Sanger sequencing. Panel A is a screenshot from IGV showing the proportion of mutant (green) and wt (brown) base calls for three urinary DNAs (surrounding wt sequence in grey). Sanger sequencing of the same three urinary DNAs is shown in panels B: sample 661 (chr5: 1,295,250 G>A), C: sample 857 (5: 1,295,228 G>A), and D: sample 576 (chr5: 1,242 & 243 G>A).

We detected 34 previously reported (COSMIC) mutations in our patient cohort. Details of these mutations and read depths at these loci are shown in S3 Table. *TERT* promoter mutations were detected in the urine of 67 out of 122 UBC patients (55%). The percentages in stage pTa, pT1 and pT2+ UBC were 56, 63 and 44%, respectively (Fig 1). *TERT* mutations were also detected 1 out of 20 non-UBC patients and in 1 out of 89 post-UBC patients. *TERT* mutations were also detected in 2 patients out of 2 with recurrent UBC. The percentage of reads supporting the presence of the mutation varied from <5% to >50% indicating that the ratio of tumour to normal DNA in the urine is quite variable. The correct detection of the *TERT* mutations was confirmed by Sanger Sequencing in several patients with >40% mutant reads (Fig 2).

**Fig 3:**
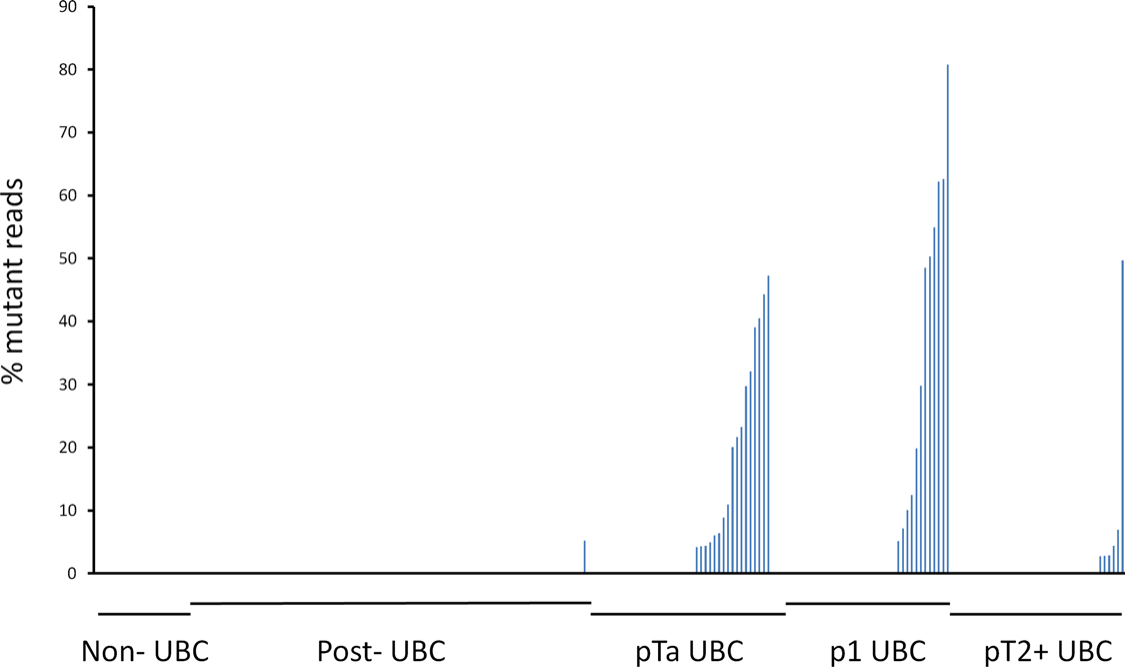
Frequency of mutant *FGFR3* reads in urinary DNA. Individual patients are listed along the x-axis as indicated and the frequency of mutant reads at comic loci is shown for each individual.

Mutations in *FGFR3* were detected in 36 urinary DNAs from patients with UBC (30%) and with the highest frequency in NMIBC (percentages in stage pTa, pT1 and pT2+ UBC were 41, 30 and 15%, respectively (Fig 3)). *FGFR3* mutations were also found in 1 post-UBC patient, and in 1 of the 2 patients with recurrent UBC. As with *TERT*, mutations were confirmed by Sanger sequencing in several samples with a high percentage of mutant reads (data not shown).

Mutations in *PIK3CA* were found in the urine of 17 patients with UBC (14%) across all stages of disease (percentages in stage pTa, pT1 and pT2+ UBC were 12, 18 and 10 respectively). A *PIK3CA* mutation was found in the urine of one post-UBC patient. Mutations in *TP53* were found in the urine of 15 patients with UBC (12%) with the highest frequency in MIBC (percentages in stage pTa, pT1 and pT2+ UBC were5, 13 and 21% respectively. Mutations in *RXRA*, *HRAS* and *KDM6A* were detected in the urine of 7, 1 and 0 patients with UBC respectively. All substitutions were non-synonymous except for 1 in *FGFR3* and those in the *TERT* promoter. The full list of mutations detected in the study is shown in S2 Table. In total 145 mutations were detected in 122 UBC patients and 4 in 110 patients without currently detectable UBC.

### Utilising mutations in urinary DNA to detect UBC

**Fig 4:**
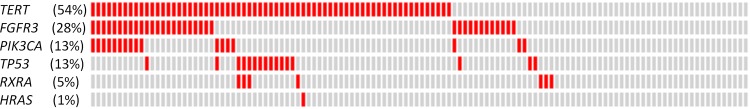
Occurrence of cosmic listed point mutations in urinary DNA from UBC patients. Each column represents an individual patient. Oncoprint representation generated at http://www.cbioportal.org/.

The distribution of the mutations in the UBC patients in this study is shown in Fig 4. If we use the presence of one or more mutations for detecting UBC, then *TERT* is the most informative (67 cancers detected), with *FGFR3* detecting an additional 12 cases, *RXRA* an additional 3 cases, and *TP53* and *PIK3CA* each detecting 2 additional cases. This approach detects 86 cancer cases out of 122, with mutations detected in 3 out of 109 patients without UBC (70% sensitivity and 97% specificity). This ‘test’ is stage agnostic with sensitivities of 70, 70 and 69% for stage pTa, pT1 and pT2+ UBC, respectively. Mutations are detected in an equivalent number of grade 1 and 2 tumours (72%) when compared to grade 3 tumours (68%), and there is no apparent relationship between tumour size or tumour number and test accuracy (S3 Fig and S4 Table, respectively). If the test threshold is increased to require mutations in 2 genes to indicate the presence of UBC, sensitivity decreases markedly to 34% but specificity increases to 100%.

## Discussion

In this study we have shown that mutations in multiple genes can be easily detected in urine DNA using a PCR and NGS-based approach. Although previous studies have used NGS to analyse *FGFR3* mutations in urinary DNA [16] and to analyse *TERT* promoter mutations in urinary DNA [17], we have now developed a method enabling a single analysis to measure both urinary biomarkers and others. It is possible to multiplex hundreds of samples for a single sequencing run, making the assay cost-effective when samples are analysed in batches: <5 USD/Euros sequencing cost per test (at the time of writing). Thus, to achieve low-costs and short turnaround times, samples would have to sent to a central testing laboratory; such infrastructure already exists in the UK and elsewhere. NGS allows the detection of a small number of tumour DNA molecules diluted in an excess of non-tumour DNA, which is not readily achieved with analogue sequencing methods or targeted approaches such as Snapshot and Massarray. It remains to be seen whether being able to identify very low levels of tumour DNA is advantageous in the surveillance setting as it is likely that tumours that are too small to detect or treat release enough mutant DNA to return a positive result (‘anticipatory diagnosis’ [7]). In our study of primary tumours, the percentage of *TERT* and *FGFR3* reads containing mutations varied continuously from undetectable to >50% of the total reads. Although highly variable between individuals, on average in UBC patients where a mutation was detected, 20% of the reads were the mutant allele suggesting that approximately 40% of urinary cell pellet DNA originates from the tumour, even in pTa UBC.

Although *TERT* and *FGFR3* are remarkably good biomarkers for UBC due to high prevalence and the focussing of point mutations at hotspots, the mutations in other genes commonly mutated in bladder cancer are not clustered in such a manner [18], and are therefore more challenging to detect—much more DNA has to be sequenced such that PCR and sequencing errors, and non-bladder cancer specific alterations, may give false positive results. Thus, although a multimarker panel will be required for high sensitivity disease detection (a more extensive panel than we have initially assessed here), the panel should be no larger than absolutely necessary, should use methods with very low error rates, and may require parallel sequencing of germline DNA. The *TERT* promoter is very GC rich and proved difficult to amplify with several different polymerases until we used KAPA2G (for both target amplification and barcode incorporation).

One of the most significant results reported here is that the assay worked for 96% of urine samples, and even on samples where the amount of DNA extracted from the urine pellets is too low to quantify by conventional means (< 1ng). The 10 samples which did not give sufficient read depth all had very low concentrations and were from patients with no detectable UBC or pTa disease. For ease, when analysing so many samples we only used 9 ul of the 100 ul of DNA extracted from 30–50 ml of urine rather than concentrating the more dilute samples and therefore it should be possible to improve the success rate in future by collecting more urine and eluting the DNA in a smaller volume. Many assays, such as those requiring bisulphite conversion, would fail on many more of the samples with low DNA content. Another important result is the very high specificity of our panel of mutations, which may make this specific ‘test’ useful in some scenarios despite the modest sensitivity; however, more likely our approach will be adapted to improve sensitivity. The third important result is the detection of 70% of pTa tumours (with high specificity against real world controls), which appears superior to most biomarkers reported to date.

We accept a number of limitations to this study. Firstly, the overall sensitivity of c.70% is considerably below the c.90% sensitivity of flexible cystoscopy [19]; therefore, this non-invasive test would need to be refined (most likely by the addition of more mutations and their amplicons) before it could be considered as an adjunct or replacement for either diagnostic or surveillance cystoscopy. Secondly, given the prevalence (versus incidence) of NMIBC, the use of such a test is more clinically relevant for detecting recurrence in the surveillance setting; however, it should be noted that the cases in our study are predominantly incident UBCs and that recurrent tumours may be more challenging to detect [20]. Finally, the majority of the 110 ‘control’ samples utilised to assess specificity were obtained from a separate cohort of patients undergoing NMIBC surveillance (‘post-UBC’, n = 89), although the 20 ‘non-UBC’ samples from the BCPP study represent subjects who had symptoms indicative of UBC (predominantly haematuria) but who were cancer-free on further urological evaluation. Future validation studies will endeavour to obtain longitudinal urine samples from a single cohort of patients, from first diagnosis through to surveillance, and to compare or evaluate the combination of NGS mutation analysis with urine cytology.

## Conclusions

We have successfully analysed multiple UBC-associated mutations in urine cell pellet DNA by NGS. The method works well on almost all urine samples, even those from which it is only possible to extract a few nanograms of DNA. The sensitivity of our selected mutation panel for detecting UBC was approximately 70% across all stages of UBC and the selected genes should be included in any such DNA-based biomarker panel for bladder cancer detection, but additional genetic or epigenetic markers [7,11,12,21,22] may be needed for a universally applicable test.

## Supporting Information

S1 Fig The dependence of PCR-NGS results on the quantity of input DNA. (PPTX)

S2 Fig Detection of varying levels of mutation *FGFR3* and *TERT* DNA in an excess of wtDNA. Each graph shows the results of PCR-NGS analyses of a series of 2-fold dilutions of mutant DNA.
(PPTX)

S3 Fig Oncoprint representation of the mutations detected in urinary DNA in patients with grade 1 & grade 2 UBC and grade 3 UBC. (PPTX)

S1 Table Primer sequences for PCR. (XLSX)

S2 Table Occurrence of mutations in urinary DNA. Data are presented as percentage of mutant reads at each locus.
(XLSX)

S3 Table Summary of mutation coordinates, consequences and read depths. (XLSX)

S4 Table Summary of tumour size and multiplicity and test accuracy. (XLSX)

## Abbreviations

NGS: next generation sequencing
UBC: urothelial bladder cancer
NMIBC: non-muscle invasive bladder cancer
MIBC: muscle-invasive bladder cancer
