# Supplementary material for: Multiplex PCR and Next Generation Sequencing for the Non-Invasive Detection of Bladder Cancer
Source: PLoS One. 2016 Feb 22;11(2):e0149756. doi: 10.1371/journal.pone.0149756 (PMC4762704; doi:10.1371/journal.pone.0149756)
Supplement: S1 Fig — (PPTX) [file pone.0149756.s001.pptx]

## Slide 1
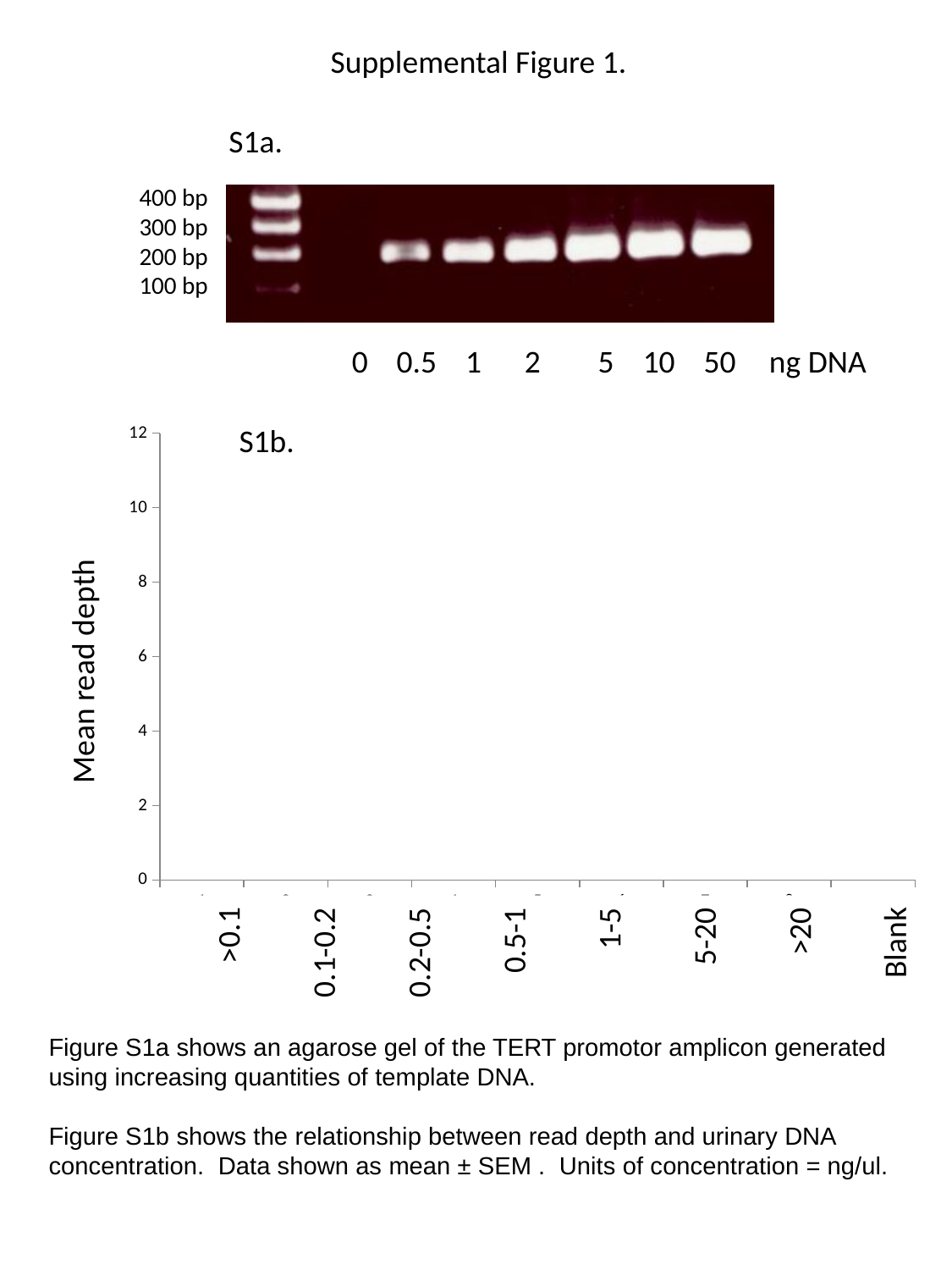

Supplemental Figure 1.
S1a.
400 bp
300 bp
200 bp
100 bp
0 0.5 1 2 5 10 50
ng DNA
### Chart
| Category | |
|---|---|>0.1
0.1-0.2
0.2-0.5
0.5-1
1-5
5-20
>20
Blank
Mean read depth
S1b.
Figure S1a shows an agarose gel of the TERT promotor amplicon generated using increasing quantities of template DNA.
Figure S1b shows the relationship between read depth and urinary DNA concentration. Data shown as mean ± SEM . Units of concentration = ng/ul.
