# Supplementary material for: Multiplex PCR and Next Generation Sequencing for the Non-Invasive Detection of Bladder Cancer
Source: PLoS One. 2016 Feb 22;11(2):e0149756. doi: 10.1371/journal.pone.0149756 (PMC4762704; doi:10.1371/journal.pone.0149756)
Supplement: S2 Fig — Each graph shows the results of PCR-NGS analyses of a series of 2-fold dilutions of mutant DNA. (PPTX) [file pone.0149756.s002.pptx]

## Slide 1
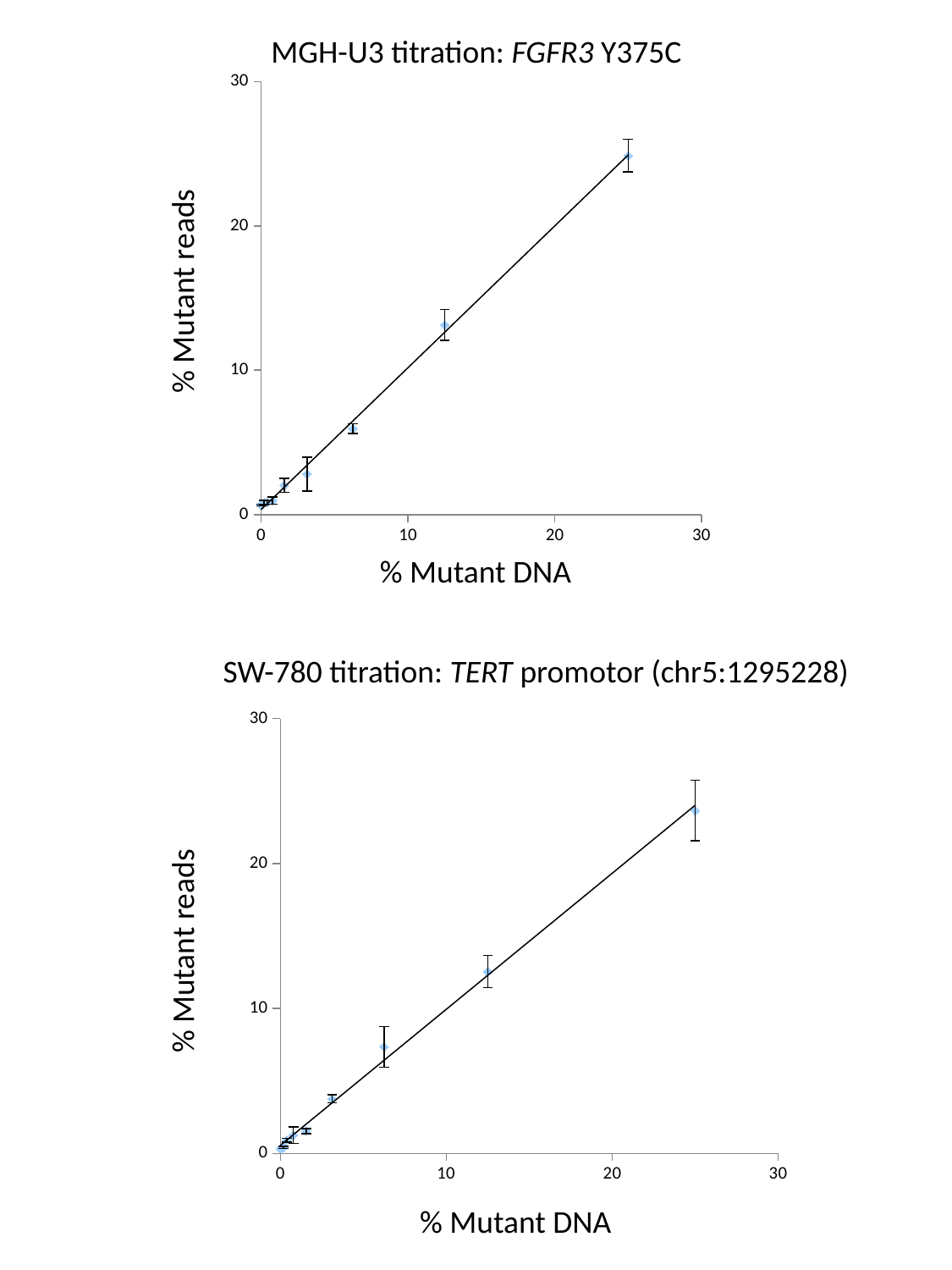

MGH-U3 titration: FGFR3 Y375C
### Chart
| Category | |
|---|---|% Mutant reads
% Mutant DNA
SW-780 titration: TERT promotor (chr5:1295228)
### Chart
| Category | MEAN |
|---|---|% Mutant reads
% Mutant DNA
