# Supplementary figures and images for: Multiplex PCR and Next Generation Sequencing for the Non-Invasive Detection of Bladder Cancer
Source: PLoS One. 2016 Feb 22;11(2):e0149756. doi: 10.1371/journal.pone.0149756 (PMC4762704; doi:10.1371/journal.pone.0149756)

## Slide 1
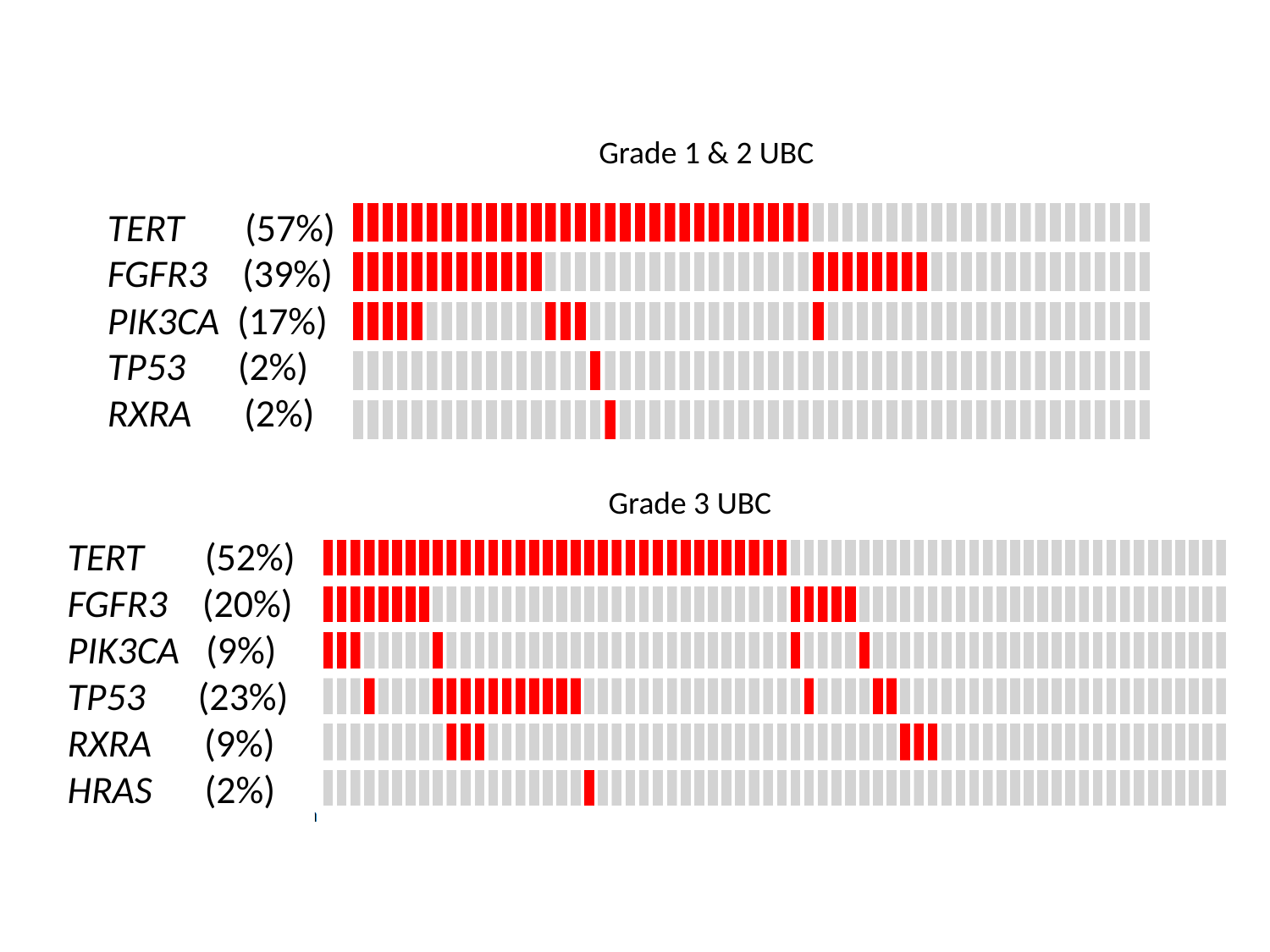

Grade 1 & 2 UBC
TERT (57%)
FGFR3 (39%)
PIK3CA (17%)
TP53 (2%)
RXRA (2%)
Grade 3 UBC
TERT (52%)
FGFR3 (20%)
PIK3CA (9%)
TP53 (23%)
RXRA (9%)
HRAS (2%)

Supplement: S3 Fig — (PPTX) [file pone.0149756.s003.pptx]
